# Reflections on the lived experience of working with limited personal protective equipment during the COVID‐19 crisis

**DOI:** 10.1111/nin.12382

**Published:** 2020-10-03

**Authors:** Kechi Iheduru‐Anderson

**Affiliations:** ^1^ School of Rehabilitation and Medical Sciences The Herbert H. and Grace A. Dow College of Health Professions Central Michigan University Mount Pleasant MI USA

**Keywords:** COVID‐19, nurse mental well‐being, nursing, pandemic, personal protective equipment, phenomenological design, workplace

## Abstract

Coronavirus disease 2019 (COVID‐19) has placed significant strain on United States’ health care and health care providers. While most Americans were sheltering in place, nurses headed to work. Many lacked adequate personal protective equipment (PPE), increasing the risk of becoming infected or infecting others. Some health care organizations were not transparent with their nurses; many nurses were gagged from speaking up about the conditions in their workplaces. This study used a descriptive phenomenological design to describe the lived experience of acute care nurses working with limited access to PPE during the COVID‐19 pandemic. Unstructured interviews were conducted with 28 acute care nurses via telephone, WebEx, and Zoom. Data were analyzed using thematic analysis. The major theme, emotional roller coaster, describes the varied intense emotions the nurses experienced during the early weeks of the pandemic, encompassing eight subthemes: scared and afraid, sense of isolation, anger, betrayal, overwhelmed and exhausted, grief, helpless and at a loss, and denial. Other themes include: self‐care, ‘hoping for the best’, ‘nurses are not invincible’, and ‘I feel lucky’. The high levels of stress and mental assault resulting from the COVID‐19 crisis call for early stress assessment of nurses and provision of psychological intervention to mitigate lasting psychological trauma.

## 1 BACKGROUND

During public health crises, health care professionals are expected to step up, be brave, and provide care and comfort to the affected. The entire nursing workforce is currently facing a significant demand, escalated by the coronavirus disease (COVID‐19) pandemic. As the COVID‐19 continues to spread rapidly throughout the United States, the demand for nurses will continue to rise, with patients overwhelming acute care hospitals and perhaps the entire U.S. health care system. Although everyone is under immense stress from trying to cope with the pandemic, nurses and other health care workers are faced with unique challenges ranging from protecting themselves, their families, and patients, to working longer hours, and in some cases, being forced to stretch their personal protective equipment (PPE) in very risky ways (American Nurses Association, 2020). Nurses are rising to the occasion. As U.S. leaders realized that the demand for PPE cannot be met, the Centers for Disease Control and Prevention (CDC) updated its guideline for the use of PPE, which permitted hospitals to amend their policies, allowing health care workers to reuse PPEs and move from patient to patient without changing their gowns or facemasks (CDC, 2020).

Although this move appears unprecedented, it is in line with the guidelines for changes in health care delivery during emergencies, when the focus is on saving as many lives as possible, and health care providers including nurses, may be expected to practice outside of the normal scope of their practice (Koenig, Lim, & Tsai, 2011; Powell, Christ, & Birkhead, 2008). These changes in standards of care were instituted by the Agency for Healthcare Research and Quality and the Office of the Assistant Secretary following the 911 terrorist attack, 2001 anthrax letter attacks, and the fears of the avian influenza pandemic in 2004 (Agency for Healthcare Research & Quality, 2005, 2007).

Powell et al. (2008) emphasized that during disasters and endemics, health care providers need to discuss any anticipated changes to the standards of care, particularly as it relates to limited resources, such as ventilators. Because the community and the public are expected to adjust to the scarcity of resources, Powell and colleagues stressed that ‘even before a patient comes to the hospital, political leaders and health officials must emphasize publicly that standards of care are and must be different in a public health disaster’ (Powell et al., 2008, p. 25). Health care providers must do whatever they can with the available resources. In a scarce resource environment, the focus of care shifts from the individual patient to optimizing outcomes for populations of patients (Chang, Backer, Bey, & Koenig, 2008; Koenig et al., 2011; Powell et al., 2008). Veenema and Toke (2007, p. 72C) underscored the protection of health care workers during crises, stating that ‘giving providers and their families personal protective equipment and instituting other measures such as staff rotation and stress management programs’ are essential to preventing burnout.

In the context of COVID‐19, while some hospitals require their staff to wear face masks at all times while onsite (Fox, 2020), others are preventing their workers from wearing face masks brought from home, with some hospital administrations even threatening their staff with disciplinary action, including termination (Ault, 2020). These conflicting policy changes and confusion have posed a different type of challenge for health care workers.

There have been several online reports of nurses and other health care providers being intimidated or reprimanded for speaking out about their working condition during the pandemic. This prompted the American Nurses Association (ANA) to respond, calling on Occupational Safety and Health Administration (OSHA) to remind employers that retaliation against health care workers for speaking out and raising concerns about their personal safety while caring for COVID‐19 patients is illegal (ANA, 2020c). The ANA reminded nurses experiencing retaliation from their employers of their right to file a whistleblower complaint online with OSHA.

As many hospitals continue to restrict the use of PPE to preserve their supply in anticipation of growing COVID‐19 cases with the rapidly evolving outbreak, many health care providers on the frontline believe that the PPE restrictions are impeding their ability to safeguard their welfare (ANA, 2020d). These policy changes presented by health care organizations are in line with the crisis capacity category described by the Institute of Medicine (2010) and the CDC (2020). ‘Crisis capacity is defined as adapting spaces, staff, and resources so that … you're doing the best you can with what you have. Staff may be asked to practice outside of the scope of their usual expertise. Supplies may have to be reused and recycled. In some circumstances, resources may become completely exhausted. Family members may be asked to provide basic patient hygiene and other aspects of care that do not require medical expertise’ (Institute of Medicine, 2010, p. 13).

Little research has examined the experiences of nurses during global, regional, or national health care crises related to disease outbreaks or natural disasters. Existing studies have focused on hospital preparation, availability of resources, and the safety of patients (Barbisch & Koenig, 2006; Karabacak, Ozturk, & Bahcecik, 2011; Ruchlewska et al., 2014; Tzeng & Yin, 2008), the education of hospital staff (Powers, 2007), emergency room nurses’ description and management during a crisis (Vasli and Dehghan‐Nayeri, 2016), and the psychological impact of disease outbreaks on hospital workers (Sun et al., 2020; Wu et al., 2009; Yin & Zeng, 2020). However, in mass casualty events and disease outbreaks, nurses may experience anxiety and personal loss (Sun et al., 2020; Veenema & Toke, 2007; Yin & Zeng, 2020).

Most studies of nurses’ experiences during a disease outbreak were focused on Asian countries due to current and previous experiences related to COVID‐19, Middle East respiratory syndrome‐coronavirus (MERS‐CoV), and human swine influenza outbreak (Khalid, Khalid, Qabajah, Barnard, & Qushmaq, 2016; Kim, 2018; Lam & Hung, 2013; Su et al., 2007; Sun et al., 2020; Yin & Zeng, 2020). A study conducted in Turkey to determine the crisis management activities and attitudes of hospital nurse managers during times of crisis, such as earthquakes and bomb explosions reported that over ‘71% percent of the nurse managers surveyed in these hospitals left resolution of crisis to the top hospital management, 64.7% noted they increased the number of the staff members, and 58.1% said they ignored crises’ (Karabacak et al., 2011, p. 323).

Crisis situations such as the one presented by COVID‐19 are a major barrier in providing optimal care as they have a strong impact on patients, their families, communities, and health care providers. During a crisis, nurses and other health care providers face various moral and ethical conflicts and dilemmas (Koenig et al., 2011; Tzeng & Yin, 2008). Patient care is significantly affected by several factors, such as stress and fatigue, workload, lack of time, demand for expertise (Kim, 2018; Lam & Hung, 2013; Mahmoudi, Mohmmadi, & Ebadi, 2013), influx of patients, experiences of health care providers, as well as level of managerial support (Hagbaghery, Salsali, & Ahmadi, 2004; Healy & Tyrrell, 2011; Kelley et al., 2004). An ANA survey of 32,174 nurses working on the frontline during the COVID‐19 crisis indicated that 74% were concerned about the lack of PPE, 58% feared for their personal safety, and 64% were extremely concerned about the safety of their friends and family (ANA, 2020d).

Considering the sparseness of empirical data on the lived experiences of nurses during crises situations, especially in the United States, this study examined the experiences of frontline nurses during the COVID‐19 crisis. Crisis is defined as an undesirable event or outcome, which includes the element of surprise or disruption of action, and is a threat to the resources and well‐being of an individual within the organization. It can have negative consequences, such as increased risk of death, delay in treatment, ignoring medical advice, and putting nurses under pressure (Vasli and Dehghan‐Nayeri, 2016). In crisis situations, important lifesaving resources, such as ‘ventilators and components, oxygen and oxygen delivery devices, intensive care unit beds (adequately staffed and equipped), health care providers, medications, etc.) are likely to be scarce’ (Koenig et al., 2011, p. 3). Similarly, during the COVID‐19 outbreak, the entire nursing workforce is facing a significant demand, which is anticipated to increase at an alarming rate.

### 1.1 Purpose

The purpose of this study was to describe the lived experience of acute care nurses working with limited access to PPE during the COVID‐19 pandemic.

### 1.2 Research question

How do registered nurses describe the lived experience of working with limited PPE during the COVID‐19 crisis?

## 2 METHODOLOGY

This qualitative descriptive phenomenological study explored the lived experiences of acute care nurses working on the frontline during the COVID‐19 disease outbreak.

### 2.1 Design: Phenomenology

Descriptive phenomenology was chosen as the design for the current study because it explored and described the participants’ everyday experiences as they lived them while working with limited PPE on the frontline of the 2020 COVID‐19 crisis. Phenomenology as a research method is dedicated to describing the structures of experience as perceived by individuals without recourse to assumptions, judgments, or presuppositions (van Manen, 2017a). It is the search for structure and essence in experience, to form a deeper understanding of the nature and meaning of everyday experience (Munhall, 2012). The focus is on providing rich textured description of the individual experiences as described by those who experience it. The role of the researcher is to describe what people experience and how they experience it (Finlay, 1999), and to understand these experiences as much as possible through the eyes of the research participants.

### 2.2 Sampling

Purposive sampling augmented with snowball sampling was used to recruit participants who met the inclusion criteria. To qualify to partake in the study, the participant was required to be a registered nurse, working in an acute care setting, or in units with diagnosed COVID‐19 patients or COVID‐19 Person Under Investigation (COVID‐19 PUI). Recruitment was done through direct email to nurses working on the frontlines known to the author, via Facebook and LinkedIn posts, posts to nursing support forums, and by word‐of‐mouth. Participants were encouraged to share recruitment flyers with their colleagues to increase the sample size.

### 2.3 Ethical considerations

The study was approved and monitored by the Central Michigan University Institutional Review Board (IRB) for the Protection of Human Subjects in Research. The IRB‐approved informed consent form was emailed to the participants for their review before scheduling the telephone interviews. Prior to each interview, verbal consent to participate in the study was audio recorded and transcribed as part of the interview. To ensure confidentiality, each participant was assigned a pseudonym (Creswell, 2012), which was used throughout the research and for data presentation. All raw data were stored in dated folders in a secured network location.

### 2.4 Data collection

Unstructured interviews were conducted over the telephone, via WebEx, or Zoom from May 15 to June 20, 2020. According to Van Manen, it is essential that the researcher asks, ‘what is the experience like, and seeks an insightful description and interpretation of the phenomena’ (van Manen, 2017b). Every participant was asked a single question: *Tell me about your experience going to work during this COVID‐19 pandemic without the guarantee of all the necessary PPE required to care for patients who have or may have COVID‐19*? This was followed by prompts and probing questions urging the participants to elaborate or further expound on something they said. Because of the COVID‐19 restrictions, all interviews were conducted from a distance by phone. All interviews were recorded, with the participants’ permission, on encrypted, password protected electronic digital recorders. Twelve of the 26 participants agreed to use the camera, webcam, or FaceTime on their phones or computers during the interviews. Audio recordings were transcribed and time stamped.

### 2.5 Data analysis

Phenomenology is focused on lived experiences, aimed at describing, not explaining, how and why meanings arise, without researcher bias (Finlay, 1999). ‘Phenomenology does not look for ‘truth’ but for the participants’ perceptions of ‘their truth’—their own experiences as they perceive them’ (Sloan & Bowe, 2014, p. 1,300). Using thematic analysis as described by Burnard, Gill, Stewart, Treasure, and Chadwick (2008), once the audio recording had been transcribed, the author familiarized herself with the data and verified its accuracy by simultaneously reading the transcript and listening to the audio recordings. During this process, any personal information, which may have been erroneously included in the interview, was deleted. All transcripts were line numbered. During the second reading of each transcript, open coding was performed by highlighting sections of the text and entering words and phrases that summarize what is being said in the text into an excel spread sheet created for this purpose.

Next, all the words and phrases from each individual interview spread sheet were compiled onto a single page. Duplicate words and phrases were deleted, and overlapping and similar categories were refined and merged to reduce the number of categories. All the interview data relevant to the research purpose were allocated to the appropriate categories, which formed the final themes and subthemes. The author consulted a colleague not involved in the study to verify the coding process, and solicit unbiased feedback (Elo et al., 2014). Finally, a report was written from the information organized in this table of findings.

### 2.6 Ensuring rigor and trustworthiness

Trust in qualitative research findings may be addressed using at least two of eight key strategies developed from Lincoln and Guba's model of trustworthiness (Creswell, 2012). Lincoln and Guba (1985) introduced the criteria of credibility, transferability, dependability, and confirmability for the assessment of rigor. For the reader to appraise transferability to other settings or populations, the author has provided justification for the research design, detailed description of the inclusion criteria, sample characteristics, and data collection and analysis methods (Hader, 2010; Maher, Hadfield, Hutchings, & de Eyto, 2018).

Bracketing, which allows one to become less assuming about another's experience, to be open, nonjudgmental and compassionate, and to present data from the perspectives of the participant rather than the researcher (Chan, Fung, & Chien, 2013) was practiced. Owing to the unprecedented nature of the COVID‐19 pandemic and its persistent broadcast on mass media, keeping a reflexive journal was very important for the author. The author chose to explore the experiences of these nurses because as a nurse who no longer worked in acute care setting, I wondered what it must be like to go to work every day during this crisis. It was important to hear directly from the nurses as they reflected on their everyday lived experiences. At times during the study interviews and data analysis, I was sometimes overwhelmed by the experiences described by these nurses. Therefore, keeping a journal was very important for me to document and explore these feelings, in order to fully represent the participant experiences rather than mine. The author also engaged with other nurse colleagues to reflect on the overall effects of the pandemic and continued to maintain a reflexive journal to elucidate evolving perceptions throughout the research process (Tufford & Newman, 2012).

Member‐checking was ensured by returning to six participants to verify the transcribed audio recordings and clarify statements made during the interviews. A summary of the findings and themes was discussed with four participants in a telephone conference call. They all confirmed that the themes accurately reflected their experiences. This respondent validation is used to ensure the dependability and credibility of qualitative studies (Elo et al., 2014; Hadi & José Closs, 2016).

## 3 FINDINGS

The sample comprised of 28 nurses, 21 women and 7 men, aged 28 to 65 years. Their level of education ranged from associate degree to master's degree in nursing. All participants worked in acute care hospital, with 22 working in hospital in the northeast, 2 in the southeast, and 4 in Midwestern United States (Table 1).

**Table 1:** Participants’ demographic characteristics (*N* = 28)

Characteristics	Values
Gender	
Male	7
Female	21
Age range (in years)	28 to 65
Experience of nursing practice (in years)	3 to 42
Highest level of nursing education	
Associate's degree in nursing (ASN)	5
Bachelor's degree in nursing (BSN)	17
Master’s degree in Nursing (MSN)	6
Unit of acute care employment	
Medical–surgical unit (Med/Surg)	11
Emergency department (ED)	6
Intensive care unit (ICU)	10

The lived experience of acute care nurses working with limited access to PPE during the COVID‐19 pandemic has been summarized into four themes. The first main theme is emotional roller coaster, which describes the intensity of the varied emotions the nurses experienced during the early weeks and months of the pandemic, encompassing the following subthemes: scared and afraid, sense of isolation, anger, felt betrayed, overwhelmed and exhausted, grief, helpless and at a loss, denial. Other main themes include: self‐care, ‘hoping for the best’, ‘nurses are not invincible’, and ‘I feel lucky’. The themes, subthemes, and participants’ exemplar quotes are displayed in Table 2.

**Table 2:** Themes and participants’ statements

Themes	Subthemes	Supporting statements
Emotional roller coaster	Scared and afraid	‘I had such fear and dread, that it is all happening again. I grew up in a country where I had experienced war, extreme hunger, disease outbreaks, and isolation. I did not want to relive that again. This whole experience has been traumatizing. I am genuinely terrified of what is going to happen a few months from now. It is a nightmare’. (Doris)
		‘I was a little scared at first, a little in denial, then when they started limiting PPE, I was terrified. I thought, surely, I am going to contract this infection, in the end I did’. (Nikki)
	Sense of isolation	‘I needed the social connection, Facebook and texting was not doing it for me. Even though I know I could get very sick, going to work and feeling needed and useful helped me deal with the crushing social isolation. I wouldn't have traded the opportunity to see people. I would have lost my mind if I couldn't leave my apartment and go to work’. (Helen)
		‘I am the only nurse in my family, they are proud of me, but they don't understand what I am going through, I cannot explain it to them. When I tried to talk to a friend, she told me it is what I signed up for when I became a nurse. But I did not sign up to work with the wrong PPE and putting myself in danger. So, when I am overwhelmed or sad, I keep it to myself. I cry in the shower or my car’. (Dianne)
	Anger	‘My employer had a duty to me, to all their employees, to provide the equipment necessary for safe delivery of service and care to the patients. They failed miserably. They can blame whomever they want, but emergency preparation should have been part of their duty to their employees. The hospital administrators are not nurses, they are not at the front line, I felt that they had no business during this difficult time making the decisions about what needed to happen. Nurses are resilient but they are not invincible’. (Noah)
		‘I was angry, I am still angry and disappointed. As usual I felt like the hospital administrators were full of bulls**t. I wanted a face to face. I wanted the hospital CEO and administrators to come to the ER, the ICU to see what is going on. I wanted them to ask me how my family was coping with my absence during this time. No one did. Why didn't they? This really is a huge failure and betrayal of trust’. (Sarah)
	Felt betrayed	‘It was an ultimate betrayal to be disciplined for protecting myself when my employer was not able to do that for me. I was holding my end of the bargain, showing up, doing my job. But I was suspended for using surgical mask from home to protect myself when they were not willing to provide for me. Why do I have to use the same mask for days? Nurses and other providers were dying from doing their job, why? (Sophia)
		‘I felt like my employers were too busy covering their butts, that they continued to lie. On television, they tell the public that their main concern is the safety of their employees, but their actions were contradictory’. (Nikki)
	Overwhelmed and exhausted	‘The barrage of information was too much. I was mentally and emotionally exhausted to take advantage of them. I am still mentally exhausted. I cried a lot. I lose my patience with minimal provocation’. (Alexie)
		‘I was tired all the time. It was very hard getting out of bed, but I pushed myself to get up and go to work. After a very long day of seeing nothing but suffering and death, I feel mentally drained’. (Priest)
	Grief	‘I used to think that nurses can overcome anything, but the death of that nurse, was devastating for me. I know people die, but…, it just hit home for me, the death of a nurse, someone you work with, and… my heart just aches’. (Sophia)
		‘I am a hugger, I hug my patients if they let me when they are suffering, I hold their hands. I never let my patients die alone if I can help it. But during this time, I could not offer comfort the way I know how, I couldn't sit with the patients as they were dying because someone else needed me. At the end of my shift, I get into my car and I weep. I weep because I feel like I am not doing enough. I am just filled with this sorrow…’ (Karen)
		‘I learned about moral distress and ethical dilemma but never really thought that I needed to worry about it much. But this crisis placed me in a situation where I worried about the ethics of what I am doing as a nurse. Being forced to reuse PPE placed me and the patients at risk. Nurses are supposed to maintain a certain standard of care, but I was not able to do that. It's a heavy burden to bear. We shouldn't have been placed in that situation’. (Kenzie)
	Helpless and at a loss	‘I was so traumatized, I suffered from debilitating anxiety after 911. I am experiencing that same thing again. Deep in my heart, I feel utterly helpless’. (Kelly)
		‘I really struggle with the ethics of what we were doing at this time. When you are knowingly putting patients under your care in danger’. (Robert)
		‘We were told it is the CDC recommendation. How can that be? I did not think that it was the right thing to do. How can it be okay to do something that a week or two ago would have gotten you disciplined or even fired? It makes you feel helpless because you really don't have a choice or say in the matter’. (Amber)
	Denial	‘I thought no, no, they can't really mean that. What? (sounding incredulous) are they crazy? (shaking her head) No way they will allow this to happen at this prestigious hospital, surely we have enough PPE’. (Nikki)
		‘It was easier for me to deny what was happening… When the president of United States said something is a hoax, you want to belief it, especially if it is something really bad, even though, deep down I know it is true. Then your job is not telling you anything… It was easier for me to tell myself that it is not that serious’. (Alexie)
Self‐care habits		‘I needed to be there for my peers. But I also had to take care of myself. I never exceeded 18 hr a day, I made sure I had a day off every six days’. (Kenny)
		‘I was overwhelmed with everything. I went into this survivor mode, trying to take care of everything and everyone. I felt that if I worked hard enough it would make things better. So, it took me a while to remind myself that I am human, that I needed care as well… You get pulled in all direction to care for others, that you forget to care for yourself’. (Jane)
Hoping for the best		‘I am a fairly new employee at my job, I had earned only 36 hr of combined paid leave. So, I really didn't have a choice but to go to work. I was reusing masks and gown, something that would have gotten me at least a warning at other times. But I prayed, washed my hands very often, and just hoped for the best’. (Flower)
		‘I had to believe that I did the best that I could do under the circumstances. I placed myself in harm's way doing what I was trained to do…I prayed for my patients and hoped that everything will be okay’. (Jane)
‘Nurses are not invincible’		‘I expected that I may be expected to work even when I am positive, but to do so with limited access to PPE, that was another level of expectation. I am not a machine; I am made of flesh and blood. If you value me, you will care for me too’. (Noah)
		‘What I realized during this time is that I was a simple commodity. I was like the toilet paper and paper towels that people were buying in large quantities. My life did not matter to my employers. I was told by my manager that I signed up for it when I chose to become a nurse. I am a nurse, I am human. I am not a machine. But my employers treated me like I was a machine. Nurses need care too’. (Sarah)
‘I feel lucky’		‘I know this may sound ridiculous, but I feel very lucky and fortunate. I don't have a wife or children, my parents live in a different state. Three nurses who worked directly with me tested positive to the virus and were out flat. They did not do anything wrong. I was lucky and I thank God for it’. (Chris)
		‘Many people are sick, people are dying. I got sick and recovered. In the grand scheme of things, I count myself very lucky at the moment’. (Mark)

### 3.1 Emotional roller coaster

Emotional roller coaster aptly described the variety of emotions experienced by the nurses during the early weeks and months of the COVID‐19 crisis in the United States. Some of these emotions continue to linger. This theme can best be summarized by this statement from Amber, a medical–surgical (med/surg)/intensive care unit (ICU) nurse:

I am scared, sad, angry, depressed, disappointed, mad as hell sometimes. Then I cry like a child when I think no one is around to hear, like in my car. I am just so angry at the government and my hospital administrators… How can they let this get to this point? We are supposed to be the wealthiest and greatest country in the world. I feel sorry for myself; my heart is broken for my dead colleagues and their families; how can you die from doing your job… Sick patients dying alone every day. You can't help them; you can't be there for them… I felt lost and for the most part helpless.

#### 3.1.1 Scared and afraid

All the participants expressed being scared and afraid of going to work when everybody else is asked to stay home, of contracting the virus at work and bringing it home to their family and friends, and of the unknown. Maggie stated,

It was really unprecedented and scary… My mom who was in her mid‐sixties came to take care of my two young children. I wondered as I was leaving if that will be the day, I contract the disease and bring it home to my family, to my mother. What will happen to my children if my mom gets sick and is not able to care for them?

Carol talked about being terrified and being afraid to go to sleep after witnessing the death of several patients and two coworkers. ‘I was terrified, I am still terrified. I was afraid to go to sleep sometimes because I thought I might get sick and die if I go to sleep’. Others were afraid of the uncertainty, the lack of reliable, and sometimes conflicting, information. Doris, who had lived through a cholera outbreak in her home country where thousands of people had died, discussed being traumatized by her previous experience and afraid to be experiencing it again. She called it a ‘nightmare’.

#### 3.1.2 Sense of isolation

The sense of isolation was profound for some of the nurses. Although they went to work and were able to see their coworkers, many were isolated from their loved ones, for fear of unknowingly infecting them with the virus even when they were negative or asymptomatic. As Karen stated, ‘I lived with my mother in‐law and other vulnerable relatives, I had to isolate myself from my family. I stayed away from my family. I stayed at a motel. I was so alone, I hurt alone’. Austin who worked long hours in the ICU took to sleeping in his car sometimes, rather than going home and infecting his family.

For some, the sense of isolation stemmed from not being able to talk to their relatives about the ‘horror’ they were experiencing at work, believing that they needed to protect them from it.

I had to remain strong and not show my family how terrified I was. The deaths, the devastations, horror of watching people die alone without their loved ones was difficult. I could not discuss that with my family. I had to pretend for their sake. [Ruth]

Others felt alone in their pain because they felt that their loved ones, who are not health care workers, would not understand what they were experiencing. Helen an emergency room nurse, who lived alone far from her family discussed how not being able to connect physically with friends after work led to ‘crushing isolation and severe depression’. For her being able to go to work helped her deal with the sense of isolation.

Because some of these nurses felt like their close relative, who are not health care providers, would be unable to understand their grief, they kept their true feelings to themselves. Therefore, close relatives did not know how to offer support, and were sometimes not able to recognize when their actions were perceived as unsupportive. In these situations, the nurses felt isolated and were not able to share their experiences with those who are closest to them.

#### 3.1.3 Anger

Anger intermingled with fear was pervasive throughout the study. Many expressed their anger toward the federal government and agencies, their employers and some for their direct supervisors and managers. Mark reported being very angry initially, stating, ‘I was very angry at my hospital, the federal government, the news media. I felt that they were making things seem worse that it really was’. Chris talked about being very angry at being forced to reuse PPE (N‐95 masks and gowns). She, like several other participants, felt that their employers did not care about them and their families. She stated,

I was angry, afraid, disappointed, all at once. Who is going to care for me, my family if I get sick? How can they ask me to make my own mask, use a scarf to protect myself? Why is the standard of care in the richest country in the world so low that as a nurse I have to make my own PPE? I am still angry. Bad leadership placed me and my loved ones at risk. I did not become a nurse to be sacrificed.

Many others expressed being angry with their hospital administrators. They felt that the administrators provided misleading information aimed at protecting their interests rather than their employees. Kasey stated, ‘This is not new, nurses have been forced to work with insufficient resources for a long time. The only difference is now, we are dying faster because of it’. Others were angry because the hospital administrators, instead of coming to their units to talk to them, made, what Kenzie described as ‘silly empty emails, shallow reassurances, and empty plans’.

#### 3.1.4 Betrayal

Many of the nurses felt betrayed by their employers’ and managers’ actions during the crisis. The betrayal was closely linked to anger arising from the lack of adequate PPE, poor communication, and disregard for their safety. For example, Jackie stated,

I was very disappointment with the hospital leaders. The hospital management were more concerned with appearances… (sounded angry). It made me so angry, then very sad. I felt like to them my life didn't matter. I felt betrayed. I know I am dispensable as an employee, but I did not have to be placed in danger because of leadership ineptitude.

For some, the sense of betrayal was linked to not being able to get tested when they felt ill or after exposure to PUI, who eventually tested positive. Some of the nurses resorted to wearing masks even at home with their families. As evidenced in this excerpt from Jane.

I felt betrayed because I couldn't get tested after exposure. I did not know if I was a danger to my patients and my family. I wore mask at work and at home. I worked hard and did my part, I felt that the system, the government betrayed me.

Other nurses expressed their sense of betrayal at not being paid when they were forced to quarantine for 14 to 21 days after exposure. Some of the nurses were forced to use their earned vacation time, while those who did not have earned time went without pay. One of the nurses, Sophia, was suspended and then reinstated after her bargaining unit intervened because she refused to care for any patient without masks, and brought her own surgical mask from home.

For Carol, the sense of betrayal came when here employer refused to pay for her hotel accommodation so she could prevent spreading the disease to her young children and elderly parents. She was told that such accommodation was for individuals from out of state. She stated, ‘I am traumatized by this experience, but I am mostly angry at the betrayal by a place I have worked in for over 15 years’.

Other nurses discussed being threatened with disciplinary action up to dismissal for talking about their experience publicly. Some received emails from their managers explicitly instructing them not to talk about their work experiences during this time. Another reason for the sense of betrayal was hospital administrators’ public assertions that they have PPE for health care workers, while the nurses were ‘reusing PPEs for weeks and forced to care for patients with other conditions not requiring PPE’. Some felt that their employers and nurse leaders were not operating in good faith. Nikki explained that the crises were understandable, many were not prepared for the impact. However, she questioned why the hospital leadership ‘was lying and deceiving their employees’, and wondered if they felt that the nurses could not ‘handle the truth’. Some raised concerns about being assigned to patients who were COVID positive and patients who were not at the same time, especially having limited access to PPE and wearing the same gown and mask the entire 12‐hr shift. Flower described it as ‘a betrayal of the nurses’ and patients’ trust’, adding, ‘I really don't know if I can trust my employers again after this’.

#### 3.1.5 Overwhelmed and exhausted

Interestingly, very few participants (three) discussed being physically exhausted. All of them discussed being ‘emotionally and mentally drained’. Many discussed being so overwhelmed with emotional and mental exhaustion that they feared going to sleep or were not able to sleep. Noah shared, ‘I feel exhausted, physically and mentally, mostly mentally. I could not shake the feeling of doom in my head… I can't explain it. I just wanted to lay down and feel nothing’. Two participants talked about giving it all up. When asked to elaborate, Simone stated,

I don't think I can go on like this. There is so much death and suffering. I am losing so many patients, and I am helpless. I have truly thought about ending it all so that I don't have to see so much suffering and feel so hopeless.

Noah, elaborating on the same, stated,

I will be honest with you. I have thought about driving my car into a tree or the ravine. I have truly contemplated ending my life. That is why I decided to participate in your study. I cannot talk about my suffering with other workers, they will judge me. They will think I am weak for feeling this way. But in all my years as a nurse, I have not witnessed so much death.

Some participants discussed being physically overwhelmed by working long hours and several days without days off for rest because nurse coworkers got sick or quit their jobs for fear of contracting the virus. One of the participants discussed being ‘overwhelmingly exhausted’, but was afraid to call out sick without being COVID positive because she had not been on the job for a long time and her manager was very critical of nurses who called out, reminding the nurses that sick calls during the COVID crisis will be considered during the annual evaluation.

Many participants discussed being overwhelmed and ‘stressed out’ with the volume of information received from work, social media, and television. Some reported being short‐tempered, cried with minimal provocation, or for ‘no apparent reason’, and ‘not being able to hold it together’. Alexie discussed being aware of important stress management strategies but not being able to use them due to mental and physical exhaustion.

#### 3.1.6 Grief

Several nurses talked about their grief. Common phrases used by many of the nurses included: ‘I am heartbroken’, ‘I can't express the sorrow I feel’, ‘My heart aches from the pain I feel’, ‘I feel so much pain in my heart’, ‘I feel nothing’, ‘I am numb with the pain I feel’, ‘I am over whelmed with sadness’, ‘It is a nightmare’, and ‘I wish it is a bad dream’. Simone stated at the onset of the interview, ‘My heart is so full of aches and pain that I am afraid if I begin to cry, I will not be able to stop’.

Overwhelmingly, grief was interwoven throughout the discussions with these nurses. Their grieving was related to loss of feeling of normalcy and not having a clear idea of when things are going to turn for the better. Many wondered if things will ever get back to normal. For instance, Maggie exclaimed during the interview, ‘I think I am in mourning’. *When asked what she meant by that, she stated,*

I feel like I have lost something, something really big. I feel like things will never be the same again, I feel like I am losing a part of myself that I will never get back. I have lost colleagues. Who will be next? Am I next? It is all just too much…

Priest described feeling a profound sense of grief when he saw the news about the nurse who died from the virus, stating,

When I read the news about nurses and doctors contracting the illness and about the nurse who died in the St. Louis area, I tried to be brave for my family, but I went into my room and cried like a child. I did not know her, but I felt like my heart was broken.

Austin, an ICU nurse, expressed grief at not being present for his family and having to work when other people were home protecting themselves. ‘I know that I signed up to be a nurse, but seeing the fear in my children's faces, broke my heart’. he went on to describe being ‘filled with such sadness’ which he sometimes could not explain. Karen expressed similar feelings of sadness but for her patients and their families.

Jackie discussed her grief in the following statement:

The pain and sorrow you feel when you learn that one of your coworkers has succumbed to this deadly virus. I was shocked and dumbfounded when I heard about her death. I felt numb, like I am in an auto pilot. I tried to be brave, but my heart was just so heavy.

Kenzie expressed profound grief about having to make decisions about which patient to give the most attention as opposed to the others. She expressed concerns about the ethical and moral implications of using one gown or N95 mask for a 12‐hr shift.

Grief was sometimes mixed with guilt for many of the nurses in this study. Several of them questioned their contributions to spreading the virus to their patients, loved ones, and even strangers despite taking extra precautions to reduce the spread. Doris whose entire family tested positive and experienced mild symptoms was especially distraught at her contribution to her family's plight. She stated, ‘I almost wiped out my entire family while trying to do my job and provide for them’. Some of the guilt associated with grief resulted from not getting sick or recovering from illness while other nurses and health care providers died from the illness.

#### 3.1.7 Helpless and at a loss

The feelings of helplessness and loss were echoed by many of the participants in several ways. For many, feeling helpless and lost stemmed from having no control over what is happening. For others, it stemmed from being torn and forced to choose between their own well‐being, that of their families, their patients, and their jobs. Some felt helpless due to the loss of previous standards of care. For Kelly, the feeling of helplessness came from having no control over what was happening. She described this feeling in the following statement:

I feel like no matter what I do, things will never be the same again. People are getting sick and dying and I cannot do anything as a nurse to stop it. Everything I know about infection control…it all changed overnight. I feel like nothing I do makes any real difference.

Robert discussed feeling like he was abandoning his patients by not being able to provide the level of care he was used to providing. Helen also struggled with the ethics of working below the accepted standard of care.

It went against everything we swore not to do. Sometimes I wondered how many people were infected because of us not having appropriate PPE. What was my contribution to the crisis? It was difficult not having control over my practice at this time. I felt like my hands were tied behind my back.

Robert's concerns were echoed by Amber who questioned the information being provided by her employer.

#### 3.1.8 Denial

Least among the nurses’ roller coaster of emotions was denial. Despite the evidence of the presence of the COVID‐19 in the United States, some of the nurses stated they were in denial. Ruth stated, ‘I guess, if I refuse to belief it, then it will not be true’. Sarah's denial was based on her belief that the United States was prepared to deal with the COVID‐19, and that what was happening in China was too far away to be a problem for the United States. She stated,

It was like an out of body experience for me. I just couldn't believe it, you know. It is not as bad as they say, it can't be. It is happening in China, I think we are better than this, we are the United States of America, surely, we are prepared for this, but we were not prepared. How can this be happening? This can't be happening. Sometimes, I think I am still in denial.

Others were in denial about the enormity of the spread. Dianne an ICU nurse in a rural community hospital stated,

I thought it will be a big city problem, like New York and Seattle. The reality really did not hit until my hospital had the first case, and then there was the news about the nurse who died from the virus. That's when it fully dawned on me. This s**t is for real.

Other nurses were in denial because they were receiving mixed messages from their employers, managers, and the government, and because it was easier to deny the reality.

### 3.2 Self‐care habits/changed how I work

Self‐care, and the lack thereof, was expressed by more than half of the participants. Some described self‐care as maintaining connections to other people, family, and friends during the difficult time. For others it meant keeping up with their routines prior to the crisis, like exercising, taking time to rest, and connecting with loved ones. Alexie stated,

I used this time to connect to all my friends and family. I live two states away from the rest of my family… I called my family almost every day, I needed to reassure them and myself that everything is okay. Talking to them kept me grounded…

Other nurses described using ‘prayer, meditation, and yoga to find balance’. Three participants talked about listening to empowering and calming podcasts while driving to work or before bed. Some dealt with their feelings by crying. Abby said, ‘when I feel like crying, I go to a quiet place to cry, then I feel better. It is cleansing for my spirit’. Kenny discussed setting boundaries and limits on how many hours he worked in a week, to prevent burnout. Some participants changed their work routine with patients, bundling patient care activities to limit exposure time while caring for COVID‐19 patient.

Some discussed not being able to ‘shut it off’ even when away from work. Watching excessive television or following the news on social media affected their sleep and increased their anxiety. Jane talked about forgetting to care for herself while caring for others. Some of the participants used some unhealthy practices, such as increased smoking, alcohol consumption, and overeating or eating ‘comfort foods’, which were not particularly healthy to deal with increased stress.

### 3.3 Hoping for the best

Hoping for the best described what most of the nurses did once they reconciled to not having control over the pandemic or the non‐availability of PPE. All the nurses in this study did what they were trained to do and hoped for the best outcome for themselves, their families, and their patients. For instance, Kasey stated,

Just have faith, do your best, and hope for the best. If it is your destiny to die from this virus, whether you go to work or not, you will die from it. It's like a mantra for me. It kept me from screaming out loud and going crazy. I went to work, did what I trained to do, and hoped for the best outcome.

Flower who has only been employed at her current hospital for a little over four months felt that she did not have a choice but to go to work stating that she did what she needed to do and ‘hoped for the best’. Kelly also expressed being hopeful stating,

We are nurses; we do what needs to be done. It is up to the employers and the government to provide us what we need to do the important work of taking care of patients and saving lives. In the situation we found ourselves with lack of adequate supply of PPE, and other things…sometimes limited IV supplies, we did our best and keep our fingers crossed.

### 3.4 Nurses are not invincible (Nurses are human too)

While many of these nurses have taken care of patients with various communicable diseases and worked with limited resources before, they expressed never having worked in situations where they lacked appropriate PPE. Several of the participants’ comments indicated that they felt that they were viewed as invincible, able to continue to operate without proper care. Some felt that their employers perceived their lives and well‐being as less important than that of their patients.

Twelve nurses in this study had eventually tested positive for COVID‐19; seven were symptomatic but did not require hospitalization. In describing their experiences, they compared it to being superheroes or being expected to act like superheroes. Nikki discussed testing positive and getting sick because of lack of PPE and for being forced to take care of patients with respiratory symptoms who had not yet tested positive for the virus. She stated,

People think that nurses are superheroes, not this nurse apparently. I got sick with the COVID‐19 two days after one of my patients tested positive. I had a two‐year‐old at home. My employer placed me and my family at risk. I wish they would treat nurses with more care.

Others were told by their employers that even if they tested positive, but remained asymptomatic, they had to continue working. Noah expressed surprise at this instruction from her unit manager, stating, ‘Nurses are often viewed as machines, unbreakable. We can be expected to be superhumanly resourceful and resilient, but in this crisis, we needed a little more caring’.

Several of the nurses talked about the need to feel supported and appreciated for what they were doing during the crisis when many around the world were sheltering in place, but they had to go to work. This is evidenced by Sophia's statement:

I am very grateful that the hospital eventually recognized the important work we were doing, that we too needed caring for. When they started providing safe transportation and meals for us, I was grateful. It made me feel like someone cared. Under the circumstances we had to work, it made a difference.

The above statement is in contrast to Abby's statement about not feeling supported by her employers and managers, comparing herself to hospital equipment, especially during the earlier days of the pandemic. She stated,

In the first three weeks of this madness, I just wanted to feel supported, I wanted to feel that my leaders and employers cared about me; I did not feel that… I felt like I was easily dispensable and placed at the same level as the hospital equipment. I seriously considered quitting, but I couldn't do that to my colleagues. Nurses just want to be valued as humans…

### 3.5 ‘I feel lucky’

The participants talked about feeling lucky. Lucky that they were not sick, were able to work and provide for their families and their patients during a difficult time. Many felt lucky to have jobs, while others expressed being lucky to not have died from the infection like some of their colleagues. As stated by Brian, an emergency room nurse,

I was scared, but mostly I felt very lucky that so far, I have been spared. No one in my family is sick and I am able to go to work as much as I can. A lot of other people were not that lucky.

This sentiment was echoed by Carol whose medical–surgical telemetry unit was converted to a COVID‐19 ICU. She stated,

Although this is a very tough time for all health care providers at the frontline, I consider myself lucky to be there to provide care to the sickest of the sick. I could have been the one lying there sick, but I am not. So, I tried not to think too much about the shortage of PPE. I say a prayer to thank God for sparing me from the disease and I offer comfort to those who need it.

Feeling lucky was expressed by several other participants, including Kasey who stated, ‘Every day when I leave to go to work, I count myself really lucky for being virus‐free and alive. Many others were not so lucky. Nurses have been infected, some have died. Many of my patients have died’.

## 4 DISCUSSION

This study aimed to describe the lived experience of acute care nurses who had to work with limited access to PPE during the COVID‐19 pandemic. Their experiences denote intense emotional turmoil described under five main themes. The fear, anger, sense of isolation, exhaustion, and helplessness are consistent with feelings described by nurses caring for COVID‐19 patients in China (Sun et al., 2020). While many Americans were following the shelter‐in‐place orders issued across the country to protect themselves from COVID‐19, tens of thousands of nurses across the United States were heading to work every day to care for patients affected by COVID‐19 and others requiring hospitalization for various ailments. The critical shortage of PPE for nurses and other health care workers placed them at risk of contracting the virus, becoming sick, and even dying. The emotional roller coaster was more pronounced during the earlier weeks of the pandemic in the United States, as also reported by Sun et al. (2020). The nurses' negative emotions were more pronounced when they first began taking care of COVID‐19 patients. O’Boyle et al. (2006) reported that nurses were overwhelmed with the workload and longer work hours because some colleagues refused to work during the crisis.

The nurses were concerned about exposing their families to the virus, which was also a concern for nurses taking care of patients during the 2003 outbreak of severe acute respiratory syndrome (SARS) in Taiwan (Lee et al., 2005), and Middle East respiratory syndrome‐coronavirus (MERS‐CoV) in South Korea. The sense of isolation was worsened with the nurses changing their home routine to protect their loved ones as was also reported by nurses caring for Ebola virus patients (Smith, Smith, Kratochvil, & Schwedhelm, 2017). Physical and mental exhaustion, and the sense of betrayal expressed by the participants has been reported in other studies (Lam & Hung, 2013; Sun et al., 2020). O’Boyle et al. (2006) reported that nurses feared they will be abandoned, have limited access to PPE, be at risk of infection, and have unmanageable numbers of patients to care for in cases of public health emergencies like COVID‐19.

With the care standards and infection control protocols changing frequently during the COVID‐19 pandemic, the nurses were confused by the conflicting information they received. These changes also created moral and ethical dilemma for the nurses. Evidence from public health literature indicates that appropriate communication of information is a major challenge during public health disasters (Powell et al., 2008; Vasli and Dehghan‐Nayeri, 2016), and poor communication and inaccurate information can weaken public trust in the government and result higher mortality rates (Choi, Kim, Moon, & Kim, 2015). The nurses in this study struggled to balance their concerns with personal safety with their ethical and moral obligation to provide quality care for their patients. This is in line with the evidence from Jiang (2020) study on the psychological impact and coping strategies of frontline medical staff in Hunan China during the outbreak of COVID‐19, as well as Kim and Choi (2016) study of nurses' experiences of care for patients with Middle East respiratory syndrome‐coronavirus in South Korea. The nurses in these two studies had great concerns for personal and family health, and experienced ethical distress with performing their job functions which entailed direct contact with potentially deadly viruses.

These nurses reported that they received conflicting information from their leaders at different levels. This is in conflict with ANA warning issued in March 2020 that a lack of PPE will increase the risk of nurses becoming ill themselves, and more equipment was necessary to mitigate potential staff shortages caused by illness and quarantines (ANA, 2020c). As reported by some of the nurses in this study, many health care organizations were not transparent with their nurses, many nurses were gagged from speaking up about the conditions in their workplaces.

Several of the nurses discussed self‐care activities, such as exercise, meditation, and listening to podcasts, used to cope with the stress of dealing with the crisis. Some mentioned avoiding watching the news. Previous studies of nurses working with patients during severe disease outbreaks have highlighted the importance of self‐care activities to improve psychological well‐being (Sun et al., 2020; Yin & Zeng, 2020). Appropriate and supportive care for nurses is critical to prevent adverse short‐ and long‐term outcomes for them and their families. Studies indicate that perceived support is an important factor for mitigating prolonged and complicated grief (Hutti et al., 2017; Kim, 2018).

In Taiwan during the 2003 SARS outbreak, ‘psychiatric services including psychoeducation, debriefing groups, a counseling hotline and individual psychotherapy’ were offered to the nurses and other health care providers to mitigate the effects of extreme stress and psychological conflict they experienced (Lee et al., 2005). None of the nurses in this study expressed receiving such services from their employers. However, the ANA Wellbeing Initiative provides resources to help nurses build resilience and take necessary steps to manage stress and overcome the trauma caused by COVID‐19 (ANA, 2020a).

Many of these nurses were hiding their emotions from their families and even their colleagues. This was described by Engler and Lasker (2000) as emotion‐focused coping strategies for dealing with grief, wherein, people who have experienced loss tend to deny the reality of the loss and do not seek the support they need to deal with it. In this study, some of the nurses, worried about talking about how they were truly feeling for fear of being perceived as weak, whereas others felt that their non‐health care‐worker families would not understand their feelings, so they hide their emotions and pretend that all is well. In Chen et al. (2020, p. e15) study, although nurse showed signs of psychological stress warranting psychological interventions, many of them ‘refused any psychological help and stated that they did not have any problems’. Billings et al. (2020) noted nurse leaders can support their nurses by being visible, ensuring access to resources to support physiological and safety needs, as well as providing evidence informed guidelines which are clear, reliable, and honest.

In the study by Chen et al. (2020), where a detailed psychological intervention plan was developed for the health care workers in a hospital in Hunan Province of China, staff were reluctant to participate. The staff were more concerned with physical needs such as the safety of their families, adequate supply of equipment and staffing, and living supplies, rest, and time for uninterrupted breaks. This is consistent with the findings from the current study. Many of the nurses, worried about their families' safety, some of the nurses took to sleeping in their cars or hotel rooms at additional expenses to safeguard their families. One of the nurses expressed gratitude when her hospital started offering them free meals while at work.

The nurses in this study did not report experiencing any stigma from the community as disease carriers. Which is in conflict with report from other studies where nurses and other health care providers reported being perceived as disease carriers and a threat to the safety of others (Maben & Bridges, 2020; Sun et al., 2020). Nurses in this study reported being angry for several reasons. Maben and Bridges (2020, p. 2,743) reported that a ‘failure to protect nursing staff adequately is causing anger and frustration, making nurses feel unsafe at work, while they are risking their own health and fearful of transmission to their families’. Another source of anger rose from the focus of inadequate access to PPE in acute and intensive care settings, making it seem that the lives of nurses and care providers in non‐acute care settings appear to matter less.

Overall, the high levels of stress and mental assault resulting from the COVID‐19 crisis calls for early stress assessment of nurses and providing psychological intervention to mitigate lasting psychological trauma. The author engaged in continued telephone communications with the two nurses who expressed wanting to hurt themselves during the interview for several weeks until they were able to secure professional psychological help.

## 5 IMPLICATIONS FOR NURSING PRACTICE

Early assessment of psychological well‐being and early intervention including frequent debriefing are important for health care professionals during a crisis. This appeared to be missing for the participants in this study. Two of the participants reported ‘wanting to end it all’, indicating suicidal ideation related to the trauma they were experiencing. The mental reserve of these nurses has been severely tested. There is an urgent need for immediate and ongoing mental care for nurses and other health care providers during this crisis.

It has been projected that the demand for nurses in critical care services in the United States will soon exceed the capabilities of the current delivery system. The COVID‐19 crisis will undoubtedly affect this demand. Understanding the nurses’ experience during this period will help develop strategies to support nurses and mitigate stressors that may cause nurses to leave the bedside. The critical shortage of PPE for nurses and other health care workers placed them at risk for contracting the virus and becoming sick and even dying. If not handled promptly and deliberately, what nurses has experienced during this crisis could become a major obstacle to recruiting additional nurses or even getting young people to join the nursing profession. This sentiment was echoed by Maben and Bridges (2020), stating that the anger and frustration caused by failure to protect nursing staff may linger after the crisis, potentially causing some nurses to leave the profession. The great strides made by the nursing profession to increase the number of racial ethnic minority nurses, may become eroded, taking the profession back decades from achieving the diversity needed to combat racial ethnic health disparities and inequities.

Moreover, findings indicate that during the early days of the COVID‐19 crisis, there may have been poor communication and missed opportunities by hospital leaders and nurse managers to safeguard the well‐being of their nurses. It is critical that health care leaders and administrations provide appropriate physical, mental, and psychological resources to support and protect health care workers.

Further, it is critical for nurse leaders and health care administrators to understand the impact of grief on the nurses. While most nurses will experience normal grief reactions in response to the COVID‐19 crisis, others may have significant, sustained, extremely intense, complex grief responses, which may negatively affect their physical and psychological well‐being. Those battered by stress may be the last to recognize it and stigma can be an obstacle to asking for help. As expressed by one of the participants, some of the nurses may not want to appear weak, put pressure on their peers, or they may fear of letting down their teams. Therefore, nurse leaders must monitor their nurses for signs of complicated grieving, such as anxiety, depressive symptoms, and signs of post‐traumatic stress disorders.

The sense of betrayal expressed by these nurses should not be brushed off. It must be addressed. There is still time for employers and nurse leaders to redeem and repair lost trust of some of their nurses. Nurse leaders and employers must respond to the needs of their nurses by using scientific evidence. Ongoing honest communication of facts and compassionate responses for the nurse's experiences must be ensured. Instead of protecting the institution, leaders must be transparent and lead with heart. Policies related to the COVID‐19 must consider the many facets of the complex issues facing the nurses instead of taking a one‐size‐fits‐all approach. The existing stigma of mental illness has not dissipated because of COVID‐19; therefore employers must do whatever they can to ensure that nurses who need help get it.

## 6 LIMITATIONS

There are several limitations to this study. First, the qualitative nature of the study limits the generalization of the findings. All the interviews were conducted from a distance through telephone or audio‐visual means, and therefor, there was limited observation of body language beyond the tone of voice. Although the study examined the lived experience of working with limited PPE during the COVID‐19 crisis, the crisis is still ongoing and many of the nurses were working in less than ideal conditions. Future studies must examine the experiences of the nurses several months and years after the crisis is under control. The experiences of others working in health care during this crisis should also be explored.

## 7 CONCLUSIONS

The COVID‐19 crisis is unprecedented. The degree to which nurses were exposed to death and experienced grief is alarming. Although there were weeks of warning of impending pandemic, health care organizations and the U.S. government failed in their duty to provide for and protect their health care workers. While many Americans socially isolated in their homes to avoid contracting the COVID‐19, nurses were heading to work, willingly exposing themselves and in some cases their families. The findings of this study indicate that many nurses across the United States now need their employers and the organizations to be present for them. Although not explicitly named in some cases, many are suffering from trauma, and sustained mental and emotional stress. They need support for their mental and emotional health. It should not be assumed that nurses would seek help if needed. Employers and leaders should preemptively offer support and in some cases should mandate that nurses speak to counselors or psychologists to promote mental and emotional well‐being. This is an important opportunity to fully recognize that nurses are invaluable but finite assets, for generations they bear inherent emotional strain on behalf of society. To mitigate the loss of currently practicing nurses which will likely worsen the projected nursing shortage, the nursing profession and health care leaders must do all they can to support the welfare of nurses during this crisis and beyond.

## References

[nin12382-bib-0001] Centers for Disease Control & Prevention . (2020). Coronavirus Disease 2019 (COVID‐19). Centers for Disease Control and Prevention. Retrieved from https://www.cdc.gov/coronavirus/2019‐ncov/hcp/respirators‐strategy/index.html

[nin12382-bib-0002] American Nurses Association . (2020d). Innovation in nursing‐ COVID‐19 survey responses. American Nurses Association. Retrieved from https://www.nursingworld.org/practice‐policy/work‐environment/health‐safety/disaster‐preparedness/coronavirus/education/innovation/

[nin12382-bib-0003] Agency for Healthcare Research and Quality . (2005). Altered standards of care in sass casualty events: Bioterrorism and other public health emergencies (No. 290–04–0010; Bioterrorism and other public health emergencies). Agency for Healthcare Research and Quality. Retrieved from https://archive.ahrq.gov/research/altstand/altstand3.htm

[nin12382-bib-0004] Agency for Healthcare Research and Quality . (2007). Mass medical care with scarce resources: A community planning guide. Retrieved from https://archive.ahrq.gov/research/mce/

[nin12382-bib-0005] American Nurses Association . (2020a). ANA ‐ The well‐being initiative. ANA. Retrieved from https://www.nursingworld.org/practice‐policy/work‐environment/health‐safety/disaster‐preparedness/coronavirus/what‐you‐need‐to‐know/the‐well‐being‐initiative/

[nin12382-bib-0006] American Nurses Association (2020b). ANA responds to coronavirus pandemic declaration. ANA. Retrieved from https://www.nursingworld.org/news/news‐releases/2020/ana‐responds‐to‐coronavirus‐pandemic‐declaration/

[nin12382-bib-0007] American Nurses Association . (2020c). ANA disturbed by reports of retaliation against nurses for raising concerns about COVID‐19 safety. ANA. Retrieved from https://www.nursingworld.org/news/news‐releases/2020/ana‐disturbed‐by‐reports‐of‐retaliation‐against‐nurses‐for‐raising‐concerns‐about‐covid‐19‐safety/

[nin12382-bib-0008] Ault, A. (2020). Amid PPE shortage, clinicians face harassment, firing for self‐care. Medscape. Retrieved from http://www.medscape.com/viewarticle/927590

[nin12382-bib-0009] Barbisch, D. F. , & Koenig, K. L. (2006). Understanding surge capacity: Essential elements. Academic Emergency Medicine, 13(11), 1098–1102. 10.1197/j.aem.2006.06.041 17085738

[nin12382-bib-0010] Billings, J. , Kember, T. , Greene, T. , Grey, N. , El‐Leithy, S. , Lee, D. , … Bloomfield, M. (2020). Guidance for planners of the psychological response to stress experienced by hospital staff associated with COVID: Early interventions. COVID Trauma Response Working Group rapid guidance. Retrieved from https://232fe0d6‐f8f4‐43eb‐bc5d‐6aa50ee47dc5.filesusr.com/ugd/6b474f_5626bd1321da4138b1b43b78b8de2b20.pdf

[nin12382-bib-0011] Burnard, P. , Gill, P. , Stewart, K. , Treasure, E. , & Chadwick, B. (2008). Analysing and presenting qualitative data. British Dental Journal, 204(8), 429–432. 10.1038/sj.bdj.2008.292 18438371

[nin12382-bib-0012] Chan, Z. C. Y. , Fung, Y. , & Chien, W. (2013). Bracketing in phenomenology: Only undertaken in the data collection and analysis process? The Qualitative Report, 18(30), 1–9.

[nin12382-bib-0013] Chang, E. F. , Backer, H. , Bey, T. A. , & Koenig, K. L. (2008). Maximizing medical and health outcomes after a catastrophic disaster: Defining a new “crisis standard of care”. (Abstract). *Western* . Journal of Emergency Medicine, 9(3), S3.

[nin12382-bib-0014] Chen, Q. , Liang, M. , Li, Y. , Guo, J. , Fei, D. , Wang, L. , … Zhang, Z. (2020). Mental health care for medical staff in China during the COVID‐19 outbreak. The Lancet Psychiatry, 7(4), e15–e16. 10.1016/S2215-0366(20)30078-X 32085839 PMC7129426

[nin12382-bib-0015] Choi, J. W. , Kim, K. H. , Moon, J. M. , & Kim, M. S. (2015). Public health crisis response and establishment of a crisis communication system in South Korea: Lessons learned from the MERS outbreak. Journal of the Korean Medical Association, 58(7), 10.5124/jkma.2015.58.7.624

[nin12382-bib-0016] Creswell, J. W. (2012). Qualitative inquiry and research design: Choosing among five approaches. Los Angeles, CA: SAGE Publications.

[nin12382-bib-0017] Elo, S. , Kääriäinen, M. , Kanste, O. , Pölkki, T. , Utriainen, K. , & Kyngäs, H. (2014). Qualitative content analysis: A focus on trustworthiness. SAGE Open, 4(1), 2158244014522633. 10.1177/2158244014522633

[nin12382-bib-0018] Engler, A. J. , & Lasker, J. N. (2000). Predictors of maternal grief in the year after a newborn death. Illness, Crisis & Loss, 8(3), 227–243. 10.1177/105413730000800302

[nin12382-bib-0019] Finlay, L. (1999). Applying phenomenology in research: Problems, principles and practice. British Journal of Occupational Therapy, 62(7), 299–306. 10.1177/030802269906200705

[nin12382-bib-0020] Fox, J. C. (2020). All Partners HealthCare employees now required to wear masks while on duty. The Boston Globe. Bostonglobe.com. Retrieved from https://www.bostonglobe.com/2020/03/22/metro/all‐mgh‐employees‐now‐required‐wear‐masks‐while‐duty/

[nin12382-bib-0021] Hader, R. (2010). Nurse leaders: A closer look. Nursing Management, 41(1), 25–29. 10.1097/01.NUMA.0000366900.80524.d4 20035198

[nin12382-bib-0022] Hadi, M. A. , & José Closs, S. (2016). Ensuring rigour and trustworthiness of qualitative research in clinical pharmacy. International Journal of Clinical Pharmacy, 38(3), 641–646. 10.1007/s11096-015-0237-6 26666909

[nin12382-bib-0023] Hagbaghery, M. A. , Salsali, M. , & Ahmadi, F. (2004). The factors facilitating and inhibiting effective clinical decision‐making in nursing: A qualitative study. BMC Nursing, 3(1), 2. 10.1186/1472-6955-3-2 15068484 PMC411049

[nin12382-bib-0024] Healy, S. , & Tyrrell, M. (2011). Stress in emergency departments: Experiences of nurses and doctors. Emergency Nurse: The Journal of the RCN Accident and Emergency Nursing Association, 19(4), 31–37. 10.7748/en2011.07.19.4.31.c8611 21877616

[nin12382-bib-0025] Hutti, M. H. , Myers, J. , Hall, L. A. , Polivka, B. J. , White, S. , Hill, J. , … Grisanti, M. M. (2017). Predicting grief intensity after recent perinatal loss. Journal of Psychosomatic Research, 101, 128–134. 10.1016/j.jpsychores.2017.07.016 28867418

[nin12382-bib-0026] Institute of Medicine . (2010). Forum on medical and public health preparedness for catastrophic events. Crisis standards of care: Summary of a workshop series. Washington, DC National Academies Press.

[nin12382-bib-0027] Jiang, Y. (2020). Psychological impact and coping strategies of frontline medical staff in Hunan between January and March 2020 during the outbreak of Coronavirus Disease 2019 (COVID‐19) in Hubei, China. Medical Science Monitor, 26, e924171. 10.12659/MSM.924171 32291383 PMC7177038

[nin12382-bib-0028] Karabacak, U. , Ozturk, H. , & Bahcecik, N. (2011). Crisis management: The activities of nurse managers in Turkey. Nursing Economic$, 29(6), 323–330. 22360107

[nin12382-bib-0029] Kelley, M. A. , Angus, D. , Chalfin, D. B. , Crandall, E. D. , Ingbar, D. , Johanson, W. , … Vender, J. S. (2004). The critical care crisis in the United States. Chest, 125(4), 1514–1517. 10.1378/chest.125.4.1514 15078767

[nin12382-bib-0030] Khalid, I. , Khalid, T. J. , Qabajah, M. R. , Barnard, A. G. , & Qushmaq, I. A. (2016). Healthcare workers emotions, perceived stressors and coping strategies during a MERS‐CoV outbreak. Clinical Medicine & Research, 14(1), 7–14. 10.3121/cmr.2016.1303 26847480 PMC4851451

[nin12382-bib-0031] Kim, J. S. , & Choi, J. S. (2016). Factors influencing emergency nurses' burnout during an outbreak of Middle East Respiratory Syndrome Coronavirus in Korea. Asian Nursing Research, 10(4), 295–299. 10.1016/j.anr.2016.10.002 28057317 PMC7104920

[nin12382-bib-0032] Kim, Y. (2018). Nurses’ experiences of care for patients with Middle East respiratory syndrome‐coronavirus in South Korea. American Journal of Infection Control, 46(7), 781–787. 10.1016/j.ajic.2018.01.012 29502886 PMC7132718

[nin12382-bib-0033] Koenig, K. L. , Lim, H. C. S. , & Tsai, S. H. (2011). Crisis standard of care: Refocusing health care goals during catastrophic disasters and emergencies. Journal of Experimental & Clinical Medicine, 3(4), 159–165. 10.1016/j.jecm.2011.06.003

[nin12382-bib-0034] Lam, K. K. , & Hung, S. Y. M. (2013). Perceptions of emergency nurses during the human swine influenza outbreak: A qualitative study. International Emergency Nursing, 21(4), 240–246. 10.1016/j.ienj.2012.08.008 23142054 PMC7118452

[nin12382-bib-0035] Lee, S. H. , Juang, Y. Y. , Su, Y. J. , Lee, H. L. , Lin, Y. H. , & Chao, C. C. (2005). Facing SARS: Psychological impacts on SARS team nurses and psychiatric services in a Taiwan general hospital. General Hospital Psychiatry, 27(5), 352–358. 10.1016/j.genhosppsych.2005.04.007 16168796 PMC7132375

[nin12382-bib-0036] Lincoln, Y. S. , & Guba, E. G. (1985). Naturalistic inquiry. Thousand Oaks, CA: Sage Publications.

[nin12382-bib-0037] Maben, J. , & Bridges, J. (2020). Covid‐19: Supporting nurses' psychological and mental health. Journal of Clinical Nursing, 29, 2742–2750. 10.1111/jocn.15307 32320509 PMC7264545

[nin12382-bib-0038] Maher, C. , Hadfield, M. , Hutchings, M. , & de Eyto, A. (2018). Ensuring rigor in qualitative data analysis: A design research approach to coding combining NVivo with traditional material methods. International Journal of Qualitative Methods, 17, 1–13. 10.1177/1609406918786362

[nin12382-bib-0039] Mahmoudi, H. , Mohmmadi, E. , & Ebadi, A. (2013). Barriers to nursing care in emergency wards. Iranian Journal of Nursing and Midwifery Research, 18(2), 145–151. 23983745 PMC3748571

[nin12382-bib-0040] Munhall, P. L. (2012). A phenomenological method. In P. Munhall (Ed.), Nursing research: A qualitative perspective (5th ed., pp. 113–176). Sudbury, MA: Jones & Bartlett Learning.

[nin12382-bib-0041] O’Boyle, C. , Robertson, C. , & Secor‐Turner, M. (2006). Nurses’ beliefs about public health emergencies: Fear of abandonment. American Journal of Infection Control, 34(6), 351–357. 10.1016/j.ajic.2006.01.012 16877103

[nin12382-bib-0042] Powell, T. , Christ, K. C. , & Birkhead, G. S. (2008). Allocation of ventilators in a public health disaster. Disaster Medicine and Public Health Preparedness, 2(1), 20–26. 10.1097/DMP.0b013e3181620794 18388654

[nin12382-bib-0043] Powers, M. F. (2007). Evaluation of hospital‐based disaster education. Journal of Emergency Nursing, 33(1), 79–82. 10.1016/j.jen.2006.10.008 17258063

[nin12382-bib-0044] Ruchlewska, A. , Wierdsma, A. I. , Kamperman, A. M. , van der Gaag, M. , Smulders, R. , Roosenschoon, B. J. , & Mulder, C. L. (2014). Effect of crisis plans on admissions and emergency visits: A randomized controlled trial. PLoS One, 9(3), e91882. 10.1371/journal.pone.0091882 24647274 PMC3960137

[nin12382-bib-0045] Sloan, A. , & Bowe, B. (2014). Phenomenology and hermeneutic phenomenology: The philosophy, the methodologies, and using hermeneutic phenomenology to investigate lecturers’ experiences of curriculum design. Quality & Quantity, 48(3), 1291–1303. 10.1007/s11135-013-9835-3

[nin12382-bib-0046] Smith, M. W. , Smith, P. W. , Kratochvil, C. J. , & Schwedhelm, S. (2017). The psychosocial challenges of caring for patients with Ebola virus disease. Health Security, 15(1), 104–109. 10.1089/hs.2016.0068 28192056

[nin12382-bib-0047] Su, T. P. , Lien, T. C. , Yang, C. Y. , Su, Y. L. , Wang, J. H. , Tsai, S. L. , & Yin, J. C. (2007). Prevalence of psychiatric morbidity and psychological adaptation of the nurses in a structured SARS caring unit during outbreak: A prospective and periodic assessment study in Taiwan. Journal of Psychiatric Research, 41(1), 119–130. 10.1016/j.jpsychires.2005.12.006 16460760 PMC7094424

[nin12382-bib-0048] Sun, N. , Wei, L. , Shi, S. , Jiao, D. , Song, R. , Ma, L. , … Wang, H. (2020). A qualitative study on the psychological experience of caregivers of COVID‐19 patients. American Journal of Infection Control, 48(6), 592–598. 10.1016/j.ajic.2020.03.018 32334904 PMC7141468

[nin12382-bib-0049] Tufford, L. , & Newman, P. (2012). Bracketing in qualitative research. Qualitative Social Work, 11(1), 80–96. 10.1177/1473325010368316

[nin12382-bib-0050] Tzeng, H. M. , & Yin, C. Y. (2008). Crisis management systems: Staff nurses demand more support from their supervisors. Applied Nursing Research, 21(3), 131–138. 10.1016/j.apnr.2006.08.003 18684406

[nin12382-bib-0051] van Manen, M. (2017a). Phenomenology and meaning attribution. Indo‐Pacific Journal of Phenomenology, 17(1), 1–12. 10.1080/20797222.2017.1368253

[nin12382-bib-0052] van Manen, M. (2017b). But is it phenomenology? Qualitative Health Research, 27(6), 775–779. 10.1177/1049732317699570 28682717

[nin12382-bib-0053] Vasli, P. , & Dehghan‐Nayeri, N. (2016). Emergency nurses’ experience of crisis: A qualitative study. Japan Journal of Nursing Science, 13(1), 55–64. 10.1111/jjns.12086 26176583

[nin12382-bib-0054] Veenema, T. G. , & Toke, J. (2007). When standards of care change in mass‐casualty events. AJN The American Journal of Nursing, 107(9), 72A–72F. 10.1097/01.NAJ.0000287515.53876.03 17721155

[nin12382-bib-0055] Wu, P. , Fang, Y. , Guan, Z. , Fan, B. , Kong, J. , Yao, Z. , … Hoven, C. W. (2009). The psychological impact of the SARS epidemic on hospital employees in China: Exposure, risk perception, and altruistic acceptance of risk. The Canadian Journal of Psychiatry, 54(5), 302–311. 10.1177/070674370905400504 19497162 PMC3780353

[nin12382-bib-0056] Yin, X. , & Zeng, L. (2020). A study on the psychological needs of nurses caring for patients with coronavirus disease 2019 from the perspective of the existence, relatedness, and growth theory. International Journal of Nursing Sciences, 7(2), 157–160. 10.1016/j.ijnss.2020.04.002 32292633 PMC7128423

